# Head phantoms for bioelectromagnetic applications: a material study

**DOI:** 10.1186/s12938-020-00830-y

**Published:** 2020-11-23

**Authors:** Alexander Hunold, René Machts, Jens Haueisen

**Affiliations:** 1 Institute of Biomedical Engineering and Informatics, Faculty of Computer Science and Automation, Technische Universität Ilmenau, 98693 Ilmenau, Germany; 2 Biomagnetic Center, Department of Neurology, Jena University Hospital, 07743 Jena, Germany

**Keywords:** Conductivity, Anisotropy, Impedance spectroscopy, EEG, TES, Agar hydrogel

## Abstract

**Background:**

Assessments of source reconstruction procedures in electroencephalography and computations of transcranial electrical stimulation profiles require verification and validation with the help of ground truth configurations as implemented by physical head phantoms. For these phantoms, synthetic materials are needed, which are mechanically and electrochemically stable and possess conductivity values similar to the modeled human head tissues. Three-compartment head models comprise a scalp layer with a conductivity range of 0.137 S/m to 2.1 S/m, a skull layer with conductivity values between 0.066 S/m and 0.00275 S/m, and an intracranial volume with an often-used average conductivity value of 0.33 S/m. To establish a realistically shaped physical head phantom with a well-defined volume conduction configuration, we here characterize the electrical conductivity of synthetic materials for modeling head compartments. We analyzed agarose hydrogel, gypsum, and sodium chloride (NaCl) solution as surrogate materials for scalp, skull, and intracranial volume. We measured the impedance of all materials when immersed in NaCl solution using a four-electrode setup. The measured impedance values were used to calculate the electrical conductivity values of each material. Further, the conductivities in the longitudinal and transverse directions of reed sticks immersed in NaCl solution were measured to test their suitability for mimicking the anisotropic conductivity of white matter tracts.

**Results:**

We obtained conductivities of 0.314 S/m, 0.30 S/m, 0.311 S/m (2%, 3%, 4% agarose), 0.0425 S/m and 0.0017 S/m (gypsum with and without NaCl in the compound), and 0.332 S/m (0.17% NaCl solution). These values are within the range of the conductivity values used for EEG and TES modeling. The reed sticks showed anisotropic conductivity with a ratio of 1:2.8.

**Conclusion:**

We conclude that agarose, gypsum, and NaCl solution can serve as stable representations of the three main conductivity compartments of the head, i.e., scalp, skull, and intracranial volume. An anisotropic conductivity structure such as a fiber track in white matter can be modeled using tailored reed sticks inside a volume conductor.
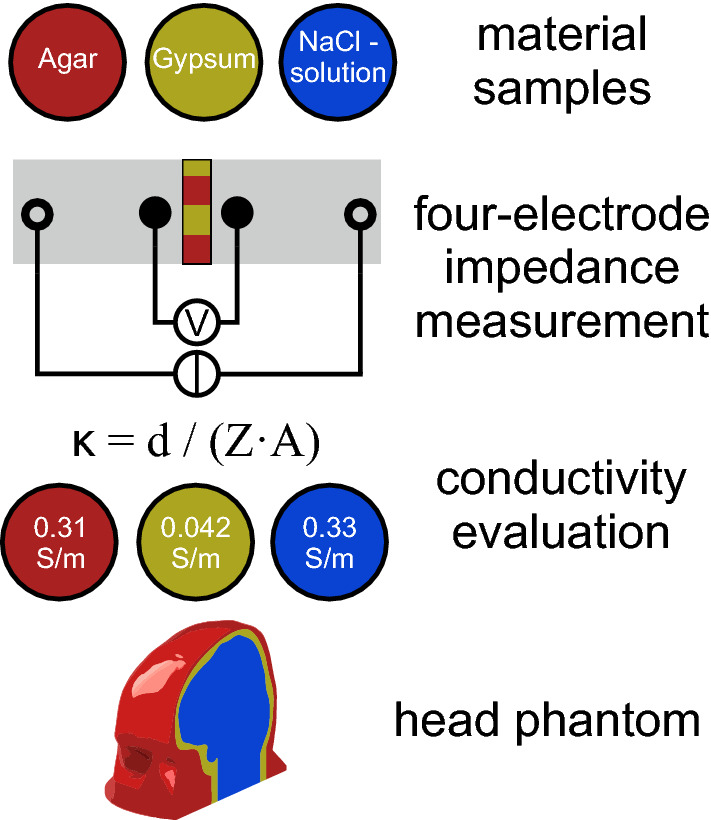

## Background

Technologies such as transcranial electric stimulation (TES), transcranial current density imaging (CDI), and neuronal source imaging based on electroencephalography (EEG) and magnetoencephalography (MEG) require methodologies for verification and validation. The evaluation of new measurement and analysis chains can be addressed by (i) computational modeling and simulation [1] and (ii) metrological inspections [2, 3]. Computational modeling and simulations provide a convenient way of assessing the above technologies. However, only metrological inspections allow the inclusion of real-world environmental influences and allow the validation of computational modeling and simulations based on ground truth.

For EEG/MEG, the spread of electromagnetic fields caused by intracranial generators is of high importance for the identification of bio-electric sources [4]. For TES, dosage considerations require exact knowledge of the spread of the electromagnetic field inside the head [5]. In both applications, the computation of the electromagnetic field requires a thorough knowledge of the volume conductor, i.e., the 3D conductivity profile within the human head. The geometry is commonly segmented from magnetic resonance imaging data sets [6] and the conductivity values are derived from literature. These values demonstrate large variations resulting from inter- and intra-individual variations [7] and differences in experimental methodology [8] and do not necessarily match the individual conductivity profiles [7, 8].

For evaluating the methodologies introduced above, using a physical phantom of the head as a volume conductor can overcome most uncertainties that occur. The geometry of the phantom is predefined by the design and manufacturing processes and the conductivity properties of the phantom materials can be measured in advance.

The skull serves as the major conductivity barrier in the human head. Consequently, there are three compartments of particular interest: scalp (soft tissue outside of the skull), skull, and intracranial volume [9]. The soft tissue compartments with higher conductivity encase the skull compartment with low conductivity. A widely used conductivity value for the intracranial volume is 0.33 S/m [10]. According to literature, the ratio for the skull-to-soft tissue conductivity ranges from 1:120 [11] to 1:5 [12]. A further feature of interest for physical representation is conductivity anisotropy, which occurs mainly in the fiber tracts of white matter. The anisotropy ratio between the longitudinal and transverse direction in white matter was varied from 1:2 to 1:100 in modeling studies [13, 14]. Nicholson found an anisotropy ratio of approximately 1:9 in impedance measurements of the white matter in cats [15].

Previous approaches used head phantoms based on post-mortem human skulls for the assessment of EEG source reconstruction procedures [16, 17], doped saline solution for the verification of TES simulations [18, 19], and human torso built from guar gum for the modeling of conductive anisotropy [20]. These phantoms incorporated saline solutions with different electrolyte concentrations to obtain different conductivity values. Interfacing multiple compartments with different saline solutions introduces concentration gradients leading to diffusion processes. The time-dependent electrolyte diffusion limits the stability of the respective conductivity configurations in such phantoms.

Our goal is to establish a stable and well-characterized setup for physical head phantom measurements. In this study, we aim to establish and characterize suitable synthetic materials that allow the fabrication of a multi-compartmental and realistically shaped human head phantom with inherently different conductivities that has a homogeneous electrolyte concentration within compartments and will not be affected by diffusion of ions between compartments. The skull, which serves as a major structural conductivity barrier is based on a material that is plastically formable during manufacturing to replicate a realistic geometry. This material should be mechanically stable enough to serve as a support structure for adjacent compartments. Similarly, the material for the scalp should provide mechanical stability, allowing the attachment of electrodes for measurements or stimulation. For the intracranial volume, we required a material that allows for easy insertion of structures for signal generation (dipoles) or measurements (electrode arrays).

## Results

### Sodium chloride solution

The 0.17% NaCl solution provided conductivities of 0.299 S/m ± 0.005 S/m (mean μ ± standard deviation σ) on the ProfiLine Cond 3310 (0.5% uncertainty) at temperatures of 20.04 °C ± 0.70 °C. With temperature compensation to 25 °C at the ProfiLine Cond 3310, conductivities of 0.333 S/m ± 0.001 S/m were measured in 0.17% NaCl solutions, in line with previously published data [21]. Considering an inner electrode distance of 25 mm and a tube diameter of 58 mm in the experimental setup (Fig. 8), the reference impedance of the 0.17% NaCl solution was calculated from the conductivity to be 31.69 Ω at 20 °C and 28.45 Ω at 25 °C (Z_25ref_). The measured impedance of the reference 0.17% NaCl solutions in the four-electrode [22] setup was 31.41 Ω ± 0.14 Ω (Z_meas_) which resulted in a conductivity value of 0.301 S/m ± 0.002 S/m (cf. Eq. 2). During these measurements, the temperature in the 0.17% NaCl solutions was 21.04 °C ± 0.18 °C (ϑ_meas_). We determine the cell constant α for the four-electrode setup to -0.026. The cell constant was calculated from Eq. 1 using *Z*_25ref_, *Z*_meas_, and *ϑ*_meas_ from the impedance measurements with only 0.17% NaCl solution in the cell.

**Fig. 1:**
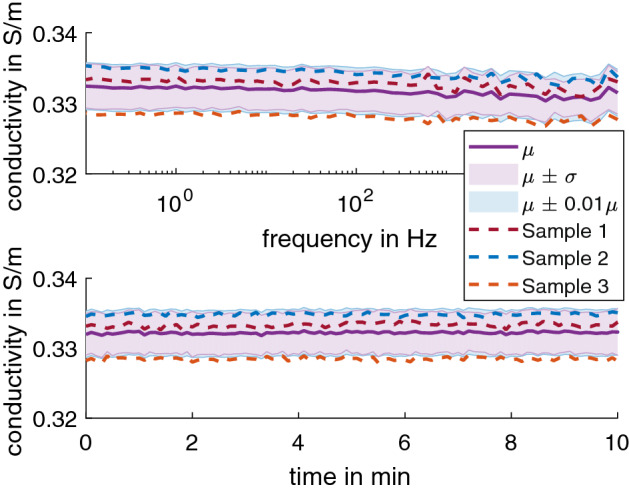
The average conductivity data (*n* = 3) at 25 °C of 0.17% NaCl solution (top) for 0.1 Hz – 100 kHz and (bottom) at 1 Hz for 10 min. The dashed line shows the average for each sample taken over three series of measurements taken with 30 min in between. The shaded regions show µ ± σ (purple) and µ ± 0.01µ (blue) for comparison

After applying Eq. 1 to adjust for the differences in the temperature at the time of measurement, the impedance of 0.17% NaCl solution was equivalent of 28.39 Ω ± 0.19 Ω at 25 °C, which is a conductivity of 0.332 S/m ± 0.003 S/m. These values were found to be consistence for frequencies of 0.1 Hz to 100 kHz (Fig. 1 top) and at 1 Hz over 10 min (Fig. 1 bottom).

### Agar hydrogel

**Fig. 2:**
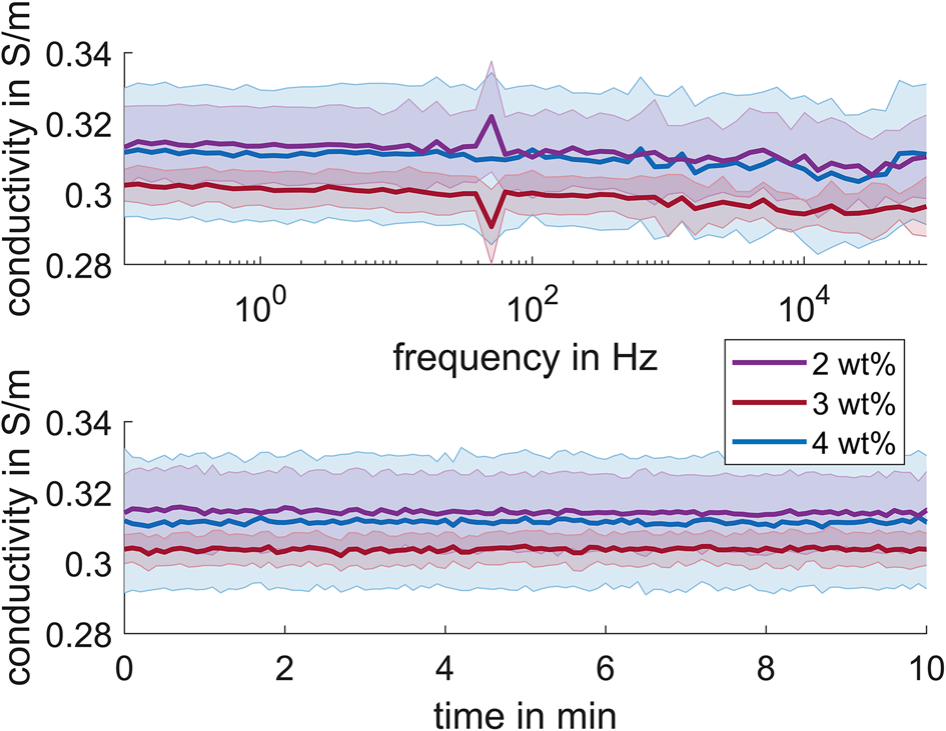
The average conductivity of 2 wt% (*n* = 3), 3 wt% (*n* = 3), and 4 wt% (*n* = 3) agar in 0.17% NaCl solution adjusted to 25 °C for (top) frequencies of 0.1 Hz to 100 kHz and (bottom) 1 Hz for 10 min. Three series of measurements were done for each sample with 30 min in between. The shaded regions show µ ± σ

The 2 wt% agar hydrogel in 0.17% NaCl solution (*n* = 3) has a conductivity of 0.284 S/m ± 0.009 S/m at 21.05 °C ± 0.23 °C, which is equivalent of 0.314 S/m ± 0.01 S/m at 25 °C (adjusted using Eq. 1). The 3 wt% agar (*n* = 3) has a conductivity of 0.272 S/m ± 0.005 S/m at 20.99 °C ± 0.19 °C, which is equivalent of 0.302 S/m ± 0.005 S/m at 25 °C (adjusted using Eq. 1). The 4 wt% agar (*n* = 3) has a conductivity of 0.281 S/m ± 0.02 S/m at 20.88 °C ± 0.41 °C, which is equivalent of 0.311 S/m ± 0.018 S/m at 25 °C (adjusted using Eq. 1). These values were found to be consistent for frequencies of 0.1 Hz to 100 kHz (Fig. 2 top) and at 1 Hz over 10 min (Fig. 2 bottom).

Even though measurements were conducted in a grounded Faraday cage, the spike at 50 Hz in the spectra (Fig. 2 top) was likely due to power-line interference.

**Fig. 3:**
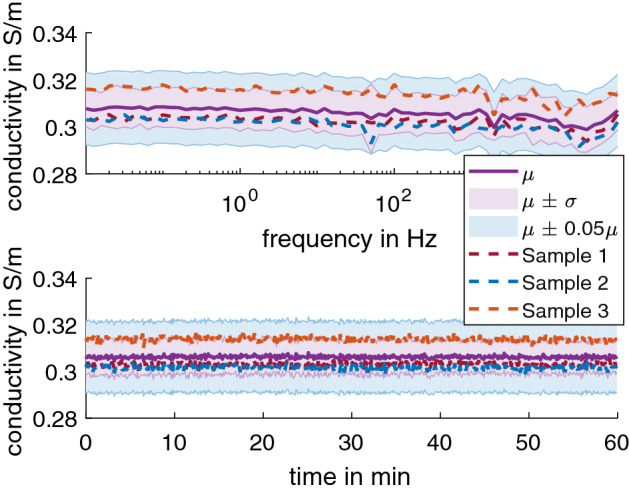
The average conductivity of 2 wt% agar in 0.17% NaCl solution adjusted to 25 °C for the (top) extended frequency range of 0.01 Hz to 100 kHz and (bottom) extended duration of 60 min at 10 Hz. The dashed line shows the data for each sample as color-coded. The shaded regions show µ ± σ (purple) and µ ± 0.05µ (blue) for comparison

The 2 wt% agar was also tested for an extended frequency range of 0.01 Hz to 100 kHz (Fig. 3 top) and duration of 60 min at 10 Hz (Fig. 3 bottom). The conductivity was found to stay consistent in this extended range, with 0.276 S/m ± 0.006 S/m at 20.76 °C ± 0.1 °C, equivalent of 0.306 S/m ± 0.007 S/m at 25 °C (adjusted using Eq. 1).

### Gypsum

**Fig. 4:**
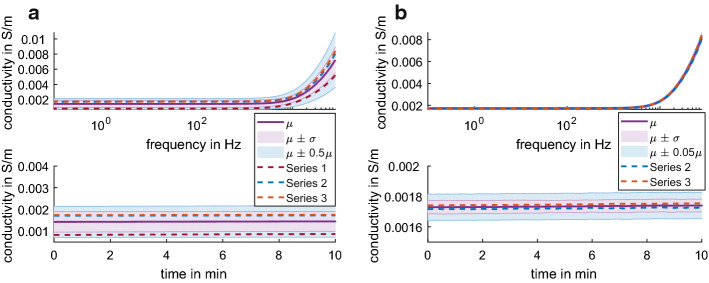
The conductivity of the gypsum sample without NaCl in the casting compound **a** average over all three series of measurements (color-coded dashed lines) in 0.17% NaCl solution adjusted to 25 °C and **b** average with the outlier (series 1) removed is shown on the right. The conductivity is shown for (top) frequencies of 0.1 Hz to 100 kHz and (bottom) 1 Hz for 10 min. The shaded regions show µ ± σ (purple) and µ ± 0.5µ (blue) for comparison on the left and µ ± 0.05µ (blue) for comparison on the right

Three series of measurements (sample immersed in 0.17% NaCl solution) were made on one sample of Stewaform gypsum without NaCl in the casting compound (Fig. 4). The sample was let rest in ambient air for 20 h between measurements.

The gypsum sample demonstrated a capacitive character with increasing conductivity for frequencies above 1 kHz, such that the phase decreased from − 8 degrees at 1 kHz to − 50 degrees at 100 kHz. For quantitative evaluations, the frequency range up to 1 kHz was considered. The sample has an average conductivity of 0.0016 S/m ± 0.0009 S/m at 25 °C for frequency up to 1 kHz. Given the dry initial condition of the gypsum, the first series of measures was an outlier with 0.0008 S/m ± 0.0001 S/m at 25 °C while the following two series of measures averaged to 0.0017 S/m ± 0.00004 S/m at 25 °C.

**Fig. 5:**
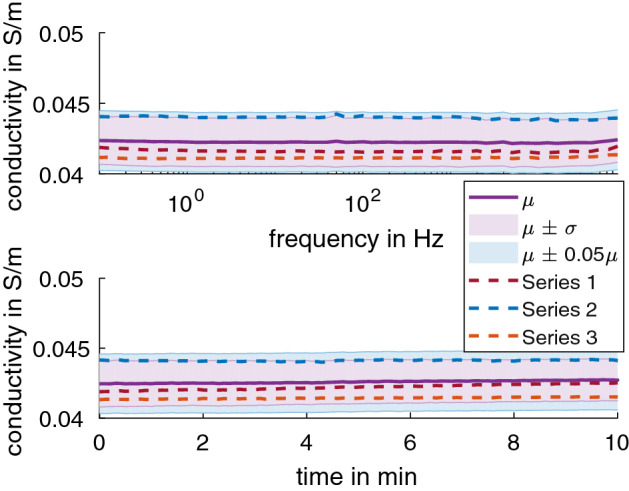
The average conductivity of the gypsum sample with NaCl in the casting compound from three series of measurements (color-coded dashed lines) in 0.17% NaCl solution adjusted to 25 °C for (top) frequencies of 0.1 Hz to 100 kHz and (bottom) 1 Hz for 10 min. The shaded regions show µ ± σ (purple) and µ ± 0.05µ (blue) for comparison

Three series of measurements (sample immersed in 0.17% NaCl solution) were made on one sample of Stewaform gypsum with NaCl in the casting compound (Fig. 5). The sample was let rest in ambient air for 20 h between measurements. This gypsum sample has a conductivity of 0.037 S/m ± 0.0012 S/m at 18.85 °C ± 0.19 °C, which is equivalent of 0.043 S/m ± 0.0015 S/m at 25 °C (adjusted using Eq. 1).

**Fig. 6:**
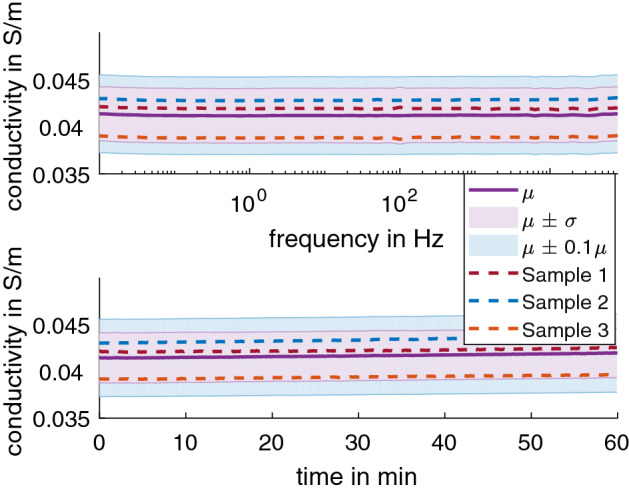
The average conductivity of gypsum with NaCl in the casting compound in 0.17% NaCl solution at 25 °C for the (top) extended frequency range of 0.01 Hz to 100 kHz and (bottom) extended duration of 60 min at 10 Hz. The dashed line shows the data for each sample as color-coded. The shaded regions show µ ± σ (purple) and µ ± 0.05µ (blue) for comparison

Three samples of gypsum with NaCl in the casting compound were tested for an extended frequency range of 0.01 Hz to 100 kHz (Fig. 6 top) and duration of 60 min at 10 Hz (Fig. 6 bottom). The conductivity was found to stay consistent in this extended range, with 0.037 S/m ± 0.002 S/m at 19.75 °C ± 0.26 °C, equivalent of 0.042 S/m ± 0.003 S/m at 25 °C (adjusted using Eq. 1).

### Conductivity anisotropy

**Fig. 7:**
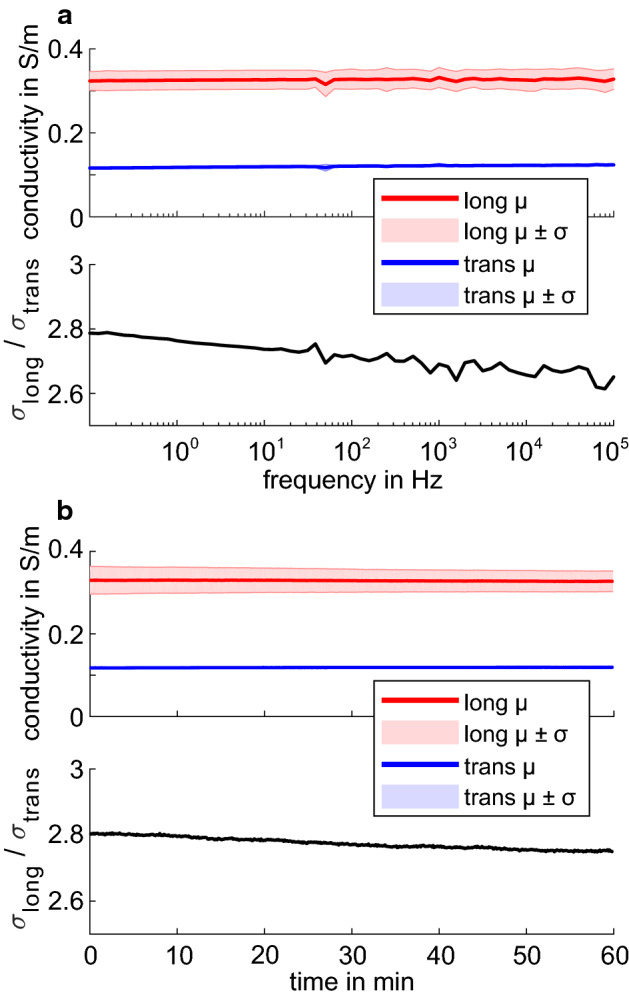
The (top) average conductivity and (bottom) anisotropy ratio of reed sticks in 0.17% NaCl solution at 25 °C for **a** frequencies of 0.1 Hz to 100 kHz and **b** duration of 60 min at 10 Hz. The shaded regions show µ ± σ for data in longitudinal (red) and transverse (blue) direction

**Fig. 8:**
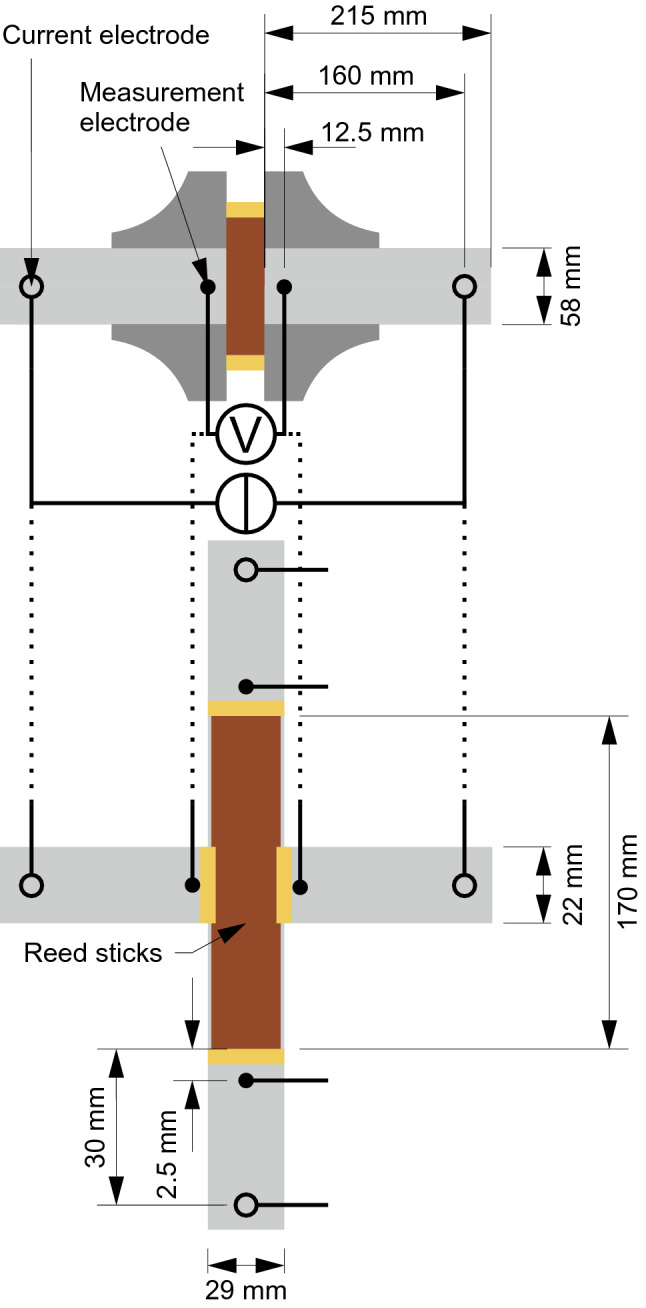
Measurement schemes for isotropic (top) and anisotropic (bottom) impedance measurements. The cell with a material sample (brown) in plastic tubes filled with NaCl solution (light gray) incorporates the paired measurement electrodes (black filled dots) and the outer electrode pair (black outlined dots) for current application, both being connected to the Gamry Reference 600 Plus impedance analyzer. Sealing rings (yellow) and POM flanges (dark gray) complete the setup (adapted from [31])

Five series of measurements (sample immersed in 0.17% NaCl solution) were made on the tube cell configuration (Fig. 8 bottom) holding 80 reed sticks for frequencies of 0.1 Hz to 100 kHz (Fig. 7a) and duration of 60 min at 10 Hz (Fig. 7b). Series of measurements were repeated with 6 h between series 1 and 2, 24 h between series 2 and 3, and series 3 and 4, and 36 h between series 4 and 5.

The conductivity in longitudinal direction was found to stay consistent, with 0.32 S/m ± 0.003 S/m in the frequency range of 0.1 Hz to 100 kHz and 0.32 S/m ± 0.02 S/m at 10 Hz over 60 min at 25 °C. The conductivity in transverse direction was found to stay consistent, with 0.12 S/m ± 0.003 S/m in the frequency range of 0.1 Hz to 100 kHz and 0.12 S/m ± 0.001 S/m at 10 Hz over 60 min at 25 °C. These conductivity differences between longitudinal and transverse direction resulted in a conductivity anisotropy ratio of 1:2.7 ± 0.05 in the frequency range of 0.1 Hz to 100 kHz and 1:2.8 ± 0.02 at 10 Hz over 60 min. Again, the spike at 50 Hz in the spectra (Fig. 7b) was likely due to power-line interference. The noise level was generally higher for frequencies above approximately 300 Hz.

## Discussion

In this study, we investigated the feasibility of establishing a realistically shaped and multi-compartmental head phantom, incorporating realistic electrolyte conductivity levels based on a 0.17% NaCl solution using agar hydrogel, gypsum, and reed sticks.

The 0.17% NaCl solution demonstrated a conductivity of 0.33 S/m at 25 °C which corresponds to the value that is widely used to model the conductivity of intracranial volume [10]. Consequently, this saline solution established the fundamental electrolyte concentration that prevailed throughout all compartments. In a closed phantom design, the saline solution itself can be used to model the intracranial volume. Interior structures for signal generation (dipoles) or measurement (electrodes) can be inserted into the aqueous solution without interfering with the structure of this compartment.

Doping the NaCl solution with agarose as a solidifying agent enabled the formation of a mechanically durable scalp layer. The conductivity value of the agar hydrogel decreased on average by 7% (2 wt%: 5.5%, 3 wt%: 9.0%, 4 wt%: 6.3%) compared to the pure NaCl solution. The variations in conductivity over time and frequency after multiple repetitions were well within µ ± 0.05µ. The average measured conductivity of 0.31 S/m at 25 °C is equivalent to 0.4 S/m at 37 °C when linearly extrapolated, which was acceptable per Burger and van Milaan [23], reporting a conductivity of 0.435 S/m at 37 °C. Further, our measured average conductivity was within the range of 0.137 S/m to 2.1 S/m summarized by McCann et al. [8]. Adapting the agar concentration in the range of 2% to 4% allows the modification of its mechanical durability without changing the conductivity to values outside of the acceptable range. With an agar concentration of 4%, applications such as EEG experiments using dry multi-pin electrodes become feasible [24].

The tested gypsum material demonstrated a considerable conductivity barrier. The two tested material configurations, with 0.17% NaCl in the casting compound and without NaCl in the casting compound, covered a wide range of skull conductivities, varying from 0.00275 S/m [11] to 0.066 S/m [12] according to literature.

After an initial soaking of the sample in NaCl solution during the first measurement, the gypsum produced without NaCl (with deionized water only) in the casting compound provided stable results within a margin of ± 5% of µ over the tested frequency range and time interval as well as across measurement repetitions. The measured average conductivity value of 0.0017 S/m at 25 °C for the gypsum without NaCl in the casting compound is equivalent to 0.0024 S/m at 36.5 °C when linearly extrapolated, which was in the same order of magnitude with skull conductivity values of 0.0038 S/m at 36.5 °C reported by Tang et al. [25], who used a very similar setup with their samples immersed in saline solution.

The gypsum samples which were produced with a 0.17% NaCl solution in the casting compound provided stable results within a margin of ± 5% of µ across multiple measurement series and was reproducible between multiple samples within a margin of ± 10% of µ. The measured average conductivity value of 0.0425 S/m at 25 °C is equivalent to 0.063 S/m at 37 °C when linearly extrapolated, which was in good accordance with skull conductivity range from 0.03 S/m to 0.08 S/m measured at 37 °C reported by Hoekema [26]. This gypsum as skull model and other phantom materials presented here established a skull-to-soft tissue conductivity ratios of 1:8, in close relation to the ratio of 1:12 reported by Oostendorp et al. [27].

The anisotropy ratio of approximately 1:3 implemented by the reed sticks is in good accordance with the ratio of 1:3 indicated in a diffusion tensor imaging data driven modeling approach [13], even though this ratio is below the white matter anisotropy ratio of 1:9 reported from earlier measurements [15]. Within a physical head phantom, reed sticks could be used to model white matter anisotropy. The sticks embody a solid model material not depending on complex support structures. Thus, they can be placed easily inside the compartment of the intracranial volume and used to model an anisotropic internal compartment.

A physical head phantom build from the materials characterized in the present study can realize the widely used approximation of the head as volume conductor comprising intracranial volume, skull, and scalp [9]. The electrolyte conductivity in all analyzed materials is based on a 0.17% NaCl solution. Consequently, the multi-compartmental phantom incorporating these materials possesses a practically stationary ion concentration. Thus, the limitation of transient conductivity configurations due to diffusion processes across compartments with varying ion concentration [2, 28, 29] can be overcome.

All materials used to manufacture the samples in this study were commercially available. Using these products and standardized production procedures, we can ensure reproducibility of the phantom production. However, all samples have been manually produced and sample properties, i.e., the area A and the thickness/length d, used in Eq. 2 had tolerances influencing the calculated conductivity values. Further, the geometry parameters of the measurement cell, reflected in the cell constant, affected the results of the temperature compensation according to Eq. 1. Impedance measurements were conducted at room temperature in a non-air-conditioned environment, and measured values were afterwards converted to the reference temperature of 25 °C [30] for comparability.

The conductivities resulting from measurements with the four-electrode setup demonstrated high reproducibility with a coefficient of variation (CV) of 0.8% and reliability with a difference of 0.2% when compared to values obtained with the ProfiLine Cond 3310.

The tested materials demonstrate consistent results when measured across several days. Consequently, head phantoms assembled with these materials have the potential of being stable throughout multi-day experiments.

## Conclusion

We investigated the applicability of NaCl solution, agar hydrogel, and gypsum for modeling intracranial volume, scalp, and skull in physical head phantoms. Agar hydrogel and gypsum are well-known moldable materials that are available at low cost and inherently mechanically stable and permeable for ions. Our measurements showed that gypsum provides a stable conductivity barrier that implements physiologically plausible skull conductivity values in contact with NaCl solution. With reed sticks, we introduced a potential material with conductive anisotropy for physical phantoms. We conclude that gypsum is suitable for producing a hollow skull compartment and can be coated with agar hydrogel realistically mimicking the scalp layer. Both materials can have varying thicknesses in the range of 1 mm to 10 mm, supporting realistic phantom construction for EEG, MEG, TES, and CDI.

## Materials and methods

### Sodium chloride solution

For reference purposes, we used NaCl (Sodium chloride ≥ 99%, Carl Roth GmbH + Co. KG, Karlsruhe, Germany) to prepare the electrolyte solutions for providing the charge carrier in the physical head phantom. The conductivity of the sodium chloride solution was tested with a ProfiLine Cond 3310 (Xylem Analytics Germany Sales GmbH & Co. KG, Weilheim, Germany).

### Agarose

We applied agarose (Agarose Broad Range, Carl Roth GmbH + Co. KG, Karlsruhe, Germany) as a solidifying agent in the NaCl solution with sufficient concentration to yield the mechanical strength of synthetic skins. We added 2 wt%, 3 wt%, and 4 wt% agarose to the heated electrolyte solution (approx. 65 °C) while stirring constantly. The milky dispersion was heated to approx. 80 °C until a clear solution emerged. The agarose electrolyte solution was kept in the liquid state, at around 65 °C, until poured into the casting mold. After cooling to room temperature, the agarose electrolyte solution formed a mechanically stable hydrogel.

### Gypsum

In gypsum, the solid crystal embodies a structural conductivity barrier. We selected Stewaform (Glorex GmbH, Rheinfelden, Germany) as casting compound in order to allow the formation of a realistic skull-shaped compartment. The Stewaform powder was mixed with either deionized water or 0.17% NaCl solution in the ratio of 2:1 to form a casting compound and poured in negative molds of the desired form and let dry at 40 °C for 2 h. To protect the gypsum structures from dissolving when coming in contact with the NaCl solution, we infiltrated the gypsum for one minute in a two-component epoxy resin XTC-3d (Smooth-On Inc., Macungie, PA, USA) at a ratio of 2:1 (epoxy resin:hardener). The gypsum was then dried again at 40 °C for 10 min.

### Reed sticks

We fit 80 reed sticks (diffusor sticks, Jörn Poppenhäger, Ottweiler, Germany) into a hollow plastic tube with an inner diameter of 29 mm and a length of 170 mm. In the middle, the tube incorporated two circular cut-outs with a diameter of 21 mm. Pellet and ring electrodes (MedCaT GmbH, Munich, Germany) were attached to the tube in 2.5 mm and 30 mm distance to the opening, respectively (cf. Fig. 8 bottom). The tube including the electrode configurations was tightly sealed with silicone and the whole volume was filled with 0.17% NaCl solution.

### Impedance measurements

We tested the electrical properties of the material samples including agar hydrogel, gypsum, and reed sticks by means of four-electrode impedance measurements at room temperature. The temperature was monitored by a Traceable Excursion-Trac (VWR International bvba, Leuven, Belgium). The impedance of the measurement cell comprising the material sample clamped between two NaCl solution compartments with a concentration of 0.17% NaCl in deionized water was measured using a Gamry Reference 600 Plus (Gamry Instruments, Warminster, PA, USA) (Fig. 8). The NaCl solution compartments held an outer pair of silver/silver chloride ring electrodes with a distance of 160 mm to the sample for impressing an electric current, and an inner pair of silver/silver chloride electrodes with 4 mm diameter for measuring the resulting potential difference. A more detailed description of the setup can be found in [31]. The reed sticks were tested in the above-mentioned double-tube configuration.

We tested the impedance of 0.17% NaCl solution (*n* = 3), agarose hydrogels with 2 wt% (*n* = 3), 3 wt% (*n* = 3), and 4 wt% (*n* = 3) agarose and each one gypsum sample with and without 0.17% NaCl solution in the casting compound. The gypsum samples were also tested three times using this procedure after they have been dried at ambient air for at least 20 h. Each series of measurements were carried out over the frequencies of 0.1 Hz to 100 kHz and for 10 min at 1 Hz. There was a 30-min pause between each series of measurements.

Further, we tested three samples of agarose hydrogels with 2 wt% agarose and one gypsum sample with 0.17% NaCl solution in the casting compound on an extended frequency range of 0.01 Hz to 100 kHz and for an extended duration of 60 min at 10 Hz. There was a 6-h pause between each series of measurements.

Simultaneous to measuring the impedances, we measured the temperature in the cell with an electrically insulated stainless steel probe connected to a Traceable® Excursion-Trac (VWR International bvba, Leuven, Belgium).

### Conductivity calculation

The experimental measurements were carried out at ambient temperatures. To compare to existing literature values, the measured values were adjusted for the temperature difference. First, the impedance of the cell containing only NaCl solution, *Z*_NaCl_, was measured to use as the reference. The impedance can then be adjusted using

1 $$Z_{25} = \frac{{Z_{{{\text{meas}}}} - Z_{{{\text{NaCl}}}} }}{{1 + \alpha \cdot (\vartheta_{{{\text{meas}}}} - 25\,\,^\circ {\text{C}})}},$$

where *Z*_25_ is the impedance adjusted to 25 °C, *Z*_meas_ is the measured impedance, ϑ_meas_ in °C is the temperature at the time of measurement, and α is the linear factor (also called cell constant). The cell constant was determined through measurements of a cell containing 0.17% NaCl solution only. The material conductivity is computed from the temperature-compensated net impedance *Z*_25_ using:

2 $$\kappa { = }\frac{d}{{Z_{{{25}}} \cdot A}},$$

where *d* is the material sample thickness/length and *A* is the surface area. The tube configuration loaded with the reed sticks was measured in longitudinal and transverse direction. For both directions, we calculated the conductivity according to (2) with *Z*_25_ obtained from (1). The conductivity anisotropy was calculated as the ratio between longitudinal and transverse conductivity.

## Data Availability

All data generated or analyzed during this study are included in this published article.
